# Aquatic Diptera in Phytotelmata of Bromeliaceae and Zingerberales

**DOI:** 10.3390/insects17030280

**Published:** 2026-03-04

**Authors:** Barbara L. Hayford, Marcella M. Jurotich, Heera Malik, Caroline S. Chaboo

**Affiliations:** 1 Division of Biological Sciences, University of Montana, Health Sciences Building, 32 Campus Dr, Missoula, MT 59812-0004, USA; 2 Department of Linguistics, University of British Columbia, 2613 West Mall, Vancouver, BC V6T 1Z4, Canada; marcella.jurotich@ubc.ca; 3 Rhithron Associates, Inc., 33 Fort Missoula Rd, Missoula, MT 59804-7203, USA; hmalik@rhithron.com; 4 Systematics Research Collections, University of Nebraska State Museum, W436 Nebraska Hall, University of Nebraska, Lincoln, NE 68588-0514, USA; cchaboo2@unl.edu

**Keywords:** biodiversity, Neotropics, larva, ecosystem, food web, insect–plant interactions

## Abstract

Phytotelmata are small, plant-held water pools that support unique assemblages of fauna and flora. Aquatic Diptera are a significant component of these microecosystems. Based on an exhaustive review of over 100 years of published literature, a global database of phytotelmata-associated aquatic Diptera with Bromeliaceae and Zingiberales (7 families) was compiled to explore diversity and ecology and identify gaps in the research. This database is designed to provide a valuable baseline and to serve as an objective, informed tool for future monitoring and research. A total of 4979 unique associations between aquatic Diptera larvae and 117 species in 26 genera of Bromeliaceae and 27 species in 10 genera across 6 families of Zingiberales were assembled in the database. These reported aquatic Diptera span 369 species, 80 genera, and 14 families, and include taxa with diverse ecological roles as detritivores, filter feeders, predators, and prey. Mosquitoes (Culicidae) had the greatest number of documented species (227), and non-biting midges (Chironomidae) had the greatest number of documented genera (26) in the dataset. Several mosquito vectors (e.g., *Anopheles*, *Aedes*, and *Culex*) also complete their life cycles in these habitats, although records of vector mosquitoes compose only 6% of the assembled data. Because phytotelmata provide year-round aquatic refugia, they sustain regional aquatic Diptera diversity yet are threatened by habitat loss and other anthropogenic pressures. Furthermore, despite the important role of phytotelmata in freshwater ecology, diversity in these ephemeral freshwater habitats remains under-documented. This comprehensive analysis of existing literature establishes the first global baseline for aquatic Diptera in phytotelmata and offers a foundation for biodiversity assessments, conservation strategies, and future ecological research.

## Simple Summary

Aquatic fly larvae are uniquely adapted to live in ephemeral, small, plant-held pools called phytotelmata. We comprehensively reviewed over 100 years of published research to document the diversity of aquatic Diptera in two of the most common plants that support phytotelmata: Bromeliaceae and Zingiberales. By extracting data from published research, we assembled a database with 4979 unique phytotelma-plant and aquatic Diptera associations that we used to identify gaps in the data and make recommendations for future research. The database is designed for use as an objective, informed tool for monitoring and research.

## 1. Introduction

Reviews and synthesis studies provide important information that can reveal major patterns and provide direction for future studies (e.g., [1]). Such studies are particularly important in contemporary biodiversity trends of the Anthropocene, characterized by widespread species losses. Freshwater macroinvertebrates, for example, are in precipitous declines worldwide [2,3]. Temporary and marginal freshwater habitats face risks due to their sporadic distribution and short-term duration (e.g., [4]). Many researchers have recognized that phytotelmata are one such aquatic microecosystem and are homes to unique assemblages of species [5,6,7,8,9,10,11]. Phytotelmata (plural; phytotelma singular), a term coined by Varga [12] from ancient Greek (phyto = plants and telma = pond), refers to ephemeral water-filled plant structures that support freshwater communities, including aquatic insects. Diptera, or true fly larvae, are one of the most diverse and numerically dominant insects in phytotelmata [8], but our understanding of their biodiversity in these abundant tropical and subtropical habitats remains poorly understood despite a century of research. Comprehensive reviews in biology are vital to identifying emergent patterns and gaps in knowledge, and we present such a review here to advance understanding of the microecosystems and inform conservation strategies.

Surveys by Picado [5] and Knab [13], along with Scott’s [14] review, initiated over a century of research on macroinvertebrates in phytotelmata. Varga [12] studied communities of water-filled leaf axils of European teasel (Caprifoliaceae: *Dipsacus*) and cited earlier works on water pools or ‘tanks’ in pitcher plants, enlarged sheath petioles, and tree holes. His synthesis attracted Albrecht Thienemann, who advanced global research on phytotelmata through his doctoral research, field expeditions, and his position at the Max-Planck Institute (Plön, Germany) [6]. The edited volume of Frank and Lounibos [15] and the review by Kitching [7] are the next major landmarks in formalized approaches to systematic study of the specialized biology of phytotelmata.

Phytotelmata have been studied as small lakes (e.g., [16]) and natural micro or mesocosms [17,18]. Kitching [7] and Greeney [8] classified phytotelmata into five and seven structural types, respectively, based on the vegetative structures that allow rainwater to pool. The vegetative parts can be leaves, bracts, fruits, seeds, tree holes, and roots (see [19]). Water in phytotelmata can vary in temperature, pH, and dissolved oxygen [7,16]. In addition, detritus and plant matter can fall into these pools, leading to nutrient accumulation, creating conditions to support small but complex food webs [20,21,22].

The biotic community within phytotelmata includes algal, bacterial, faunal, and viral life [23], and the aquatic communities are structured by species’ ability to colonize and survive within the aquatic, semi-aquatic, and drier areas of the container [24]. Despite their small size, a single phytotelma habitat may host thousands of invertebrates and many species, some of which are obligate or facultative and endemic to the habitat and region [25,26,27]. These communities may be viewed as metacommunities [17,18] or as microcosms and ecological islands (e.g., [28]), and this quality has implications for ecological and evolutionary research.

Insects dominate phytotelma invertebrate assemblages. Greeney [8] documented 70 species from 11 orders of insects, with Diptera, Coleoptera, and Odontata being the most common orders encountered. Frank and Lounibos [9] noted that larvae and pupae of aquatic Diptera were one of the most encountered groups in their overview of all insect taxa associated with bromeliad phytotelmata. Most reviews address insect communities in bromeliad-formed phytotelmata, but Siefert [29] reviewed insects from *Heliconia* (Zingiberales: Heliconiaceae), focusing on the plant structures, creation of the phytotelmata environment, insect life history, habitat succession, and basic ecology. He noted that while *Heliconia* insect communities have often been studied, published research on aquatic Diptera in *Heliconia* and other Zingiberales lags behind similar research in Bromeliaceae.

**Figure 1:**
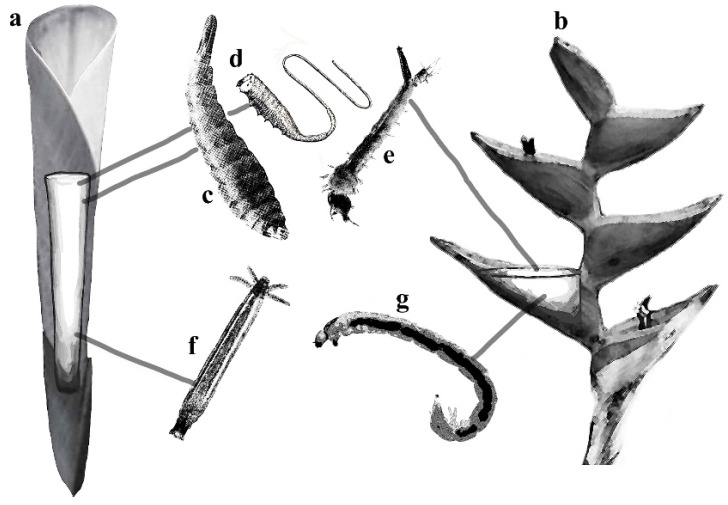
Examples of adaptations allowing aquatic Diptera to live in ephemeral habitats, such as phytotelmata with relative position in the water column indicated by gray lines: (**a**)—*Heliconia* leaf roll with stylized tank phytotelmata; (**b**)—*Heliconia* bracts with stylized phytotelmata; (**c**)—Stratiomyidae larva with hydrofuge; (**d**)—Syrphidae larva with “tail” allowing for breathing atmospheric oxygen; (**e**)—Culicidae larva with hydrofuge; (**f**)—Limoniidae larva with anal gills; (**g**)—Chironomidae larva with anal gills. *Heliconia* leaf roll and bracts modified from photos taken by C.S. Chaboo.

Aquatic Diptera are taxonomically (~46,000 species) and ecologically [30,31,32] diverse, and they are often the numerically dominant invertebrates in phytotelmata communities [25,33,34,35,36,37,38]. Thus, our review focuses on this important group of aquatic insects. Diptera are a large component of global aquatic insect diversity [39], are locally prevalent, and are ecologically significant. Aquatic Diptera are vitally important in their habitats, functioning as detritivores, predators, and prey [40]. Furthermore, adults of aquatic Diptera transport energy from the aquatic to terrestrial ecosystems [41], and some are pollinators as adults (e.g., [42]). Mosquitoes (Culicidae) are particularly important aquatic Diptera as the adults are renowned disease vectors [43,44,45,46,47,48]. Immature stages of aquatic Diptera have diverse respiratory strategies (e.g., cutaneous respiration and air breathing through respiratory siphons) that allow them to live in aquatic habitats that may have low concentrations of dissolved oxygen (Figure 1) [49,50]. Thus, some aquatic Diptera are not limited by poorly oxygenated habitats, such as those found in ephemeral pools, such as phytotelmata.

Due to their importance as vectors of disease [43,44,45,46,47,48], research on Culicidae has largely focused on their epidemiological significance and prompted extensive surveys, including habitats like phytotelmata (e.g., [51,52]). This emphasis likely biases data from phytotelmata due to the disproportionate attention to Culicidae, relative to other Dipteran families. The volume of papers on Culicidae from phytotelmata may obscure the fact that vector species may be rare in phytotelma habitats [9]. Notably, only three mosquito genera (about 2.5% of genus-level diversity) transmit diseases [53], raising questions about their perceived dominance compared to other aquatic Diptera in phytotelma habitats.

**Figure 2:**
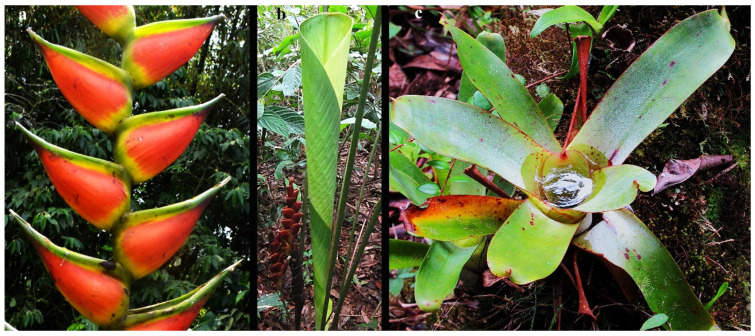
Examples of Bromeliaceae and Zingiberales: (**a**) Zingiberales, Heliconiaceae, *Heliconia*; (**b**) Zingiberales, Heliconiaceae, *Heliconia*, (**c**) Bromeliaceae. All photos taken in Trinidad, Trinidad and Tobago by C.S. Chaboo.

Phytotelmata have been documented in at least 29 families [54]. Aquatic Diptera have been reported in other phytotelmata (e.g., tree holes, [1]), but those of Bromeliaceae and Zingiberales (Figure 2) are the most extensively studied. These two plant groups are the largest taxonomically of phytotelma plants, contributing to their prominence in research [11]. Our own ongoing field-based inventories continue to expand our understanding of these microecosystems [19,55,56,57]. Accordingly, the focus of our review is on aquatic Diptera in Bromeliaceae and Zingiberales phytotelmata.

Zingiberales is a monocotyledonous order with over 2000 species classified in eight families—Cannaceae, Costacaceae, Heliconiaceae, Lowiaceae, Marantaceae, Musaceae, Strelitziaceae, and Zingiberaceae [58]. Kitching [7] considered leaf rolls and bracts in Zingiberales as one type of phytotelmata ‘axil waters’. According to generally accepted plant terminology, neither structure is axillary. Immature leaves unfurl slowly as a long rolled-up tube, with the leaf blade folded in a tight multi-layered wall around a central cone into which rainwater collects. Once fully open, the central cone disappears, and the mature leaf cannot retain a water pool. In contrast, the cup-shaped floral bracts are far smaller and hold a small volume of water around the flower bases. These two types of Zingiberales phytotelmata have been studied extensively, particularly in *Heliconia* L. in Venezuela [20,28,59].

Poales is a monocot order of over 24,000 species in 14 families [60]. Among them, Bromeliaceae (80 genera, ~3000 species) [61] are notable, valued as ornamentals, for fibers, and foods (e.g., pineapple). Bromeliads have a distinct growth form, with a rosette of leathery leaves; the leaf bases overlap and fit tightly at the base. The open vase-like form impounds rainwater and debris basally, thus the common name ‘tank bromeliad’. This pool buffers desiccation, provides habitat to other organisms, and provides nutrients to the plant [7]. The rigid leaves further compartmentalize the pool, possibly creating microhabitats within the phytotelma ecosystem. Since the earliest study [5], research on bromeliad phytotelmata has evolved to a sophisticated level (see [9]), including experimental manipulations of artificial bromeliads (e.g., [21]).

Global biodiversity declines threaten the habitats of Bromeliaceae and Zingiberales. The plants are keystone elements in their habitats, so threats to them also threaten all the organisms, including aquatic Diptera, that rely on these habitats. Deforestation, drought, hurricanes, and invasive species are all significant threats [62,63,64,65]. Researchers must understand the scope of documented diversity of aquatic Diptera from phytotelmata at a global scale to identify patterns and knowledge gaps before too much habitat is lost. Thus, our review of >100 years of publications on aquatic dipteran larvae in phytotelmata can provide a valuable baseline and an objective informed tool for future monitoring and research.

Frank and Curtis’ [66] extensive review of bromeliad mosquitoes (Diptera: Culicidae) provided the countries, geographic range, and phytotelmata habitat type for taxa they extracted from the literature. Following this format, our goals are (1) to develop a global database based on published research containing unique, georeferenced associations of aquatic Diptera and phytotelmata taxa; (2) to summarize/analyze estimates of dipteran biodiversity in phytotelmata from Bromeliaceae and Zingerberales; and (3) to identify gaps in aquatic Diptera taxa and geographic gaps in phytotelmata and make recommendations for future research.

## 2. Methods

### 2.1. Data Assembly

Data for our global database on aquatic Diptera in phytotelmata of Bromeliaceae and Zingiberales were compiled from peer-reviewed publications retrieved primarily through the Web of Science (up to 2025) and supplemented with articles found in the Florida Council of Bromeliad Societies Database, Google Scholar, and PubMed. The literature search spanned from inception in 2017, initially as part of a study by Hayford et al. [57], up to August 2025. The following search string was used: Bromeliaceae, bromeliad, Chironomidae, Ceratopogonidae, Culicidae, Diptera, Costaceae, *Heliconia*, Heliconiaceae, Musaceae, *Musa*, Marantaceae, Strelitziaceae, Zingiberaceae, phytotelmata, phytotelm, Zingiberales, Psychodidae, Tipulidae, Stratiomyidae, and Syrphidae, separately or in combination or with an asterix (e.g., phytotelma* or chironomid*). Searches included common and obscure names of aquatic Diptera to maximize coverage. We targeted only larval and pupal associations, reflecting our emphasis on the aquatic habitat. We excluded reports of adult Diptera or ambiguous accounts of Diptera where the life stage was unclear; however, that was not always possible, for example, if the life stage was not indicated in the literature.

Following the initial retrieval of articles, an extensive review of their references yielded more publications for inclusion in our database. Research topics ranged from ecology to surveys. We manually and digitally screened extensive printed and digitized records of Central and South American Culicidae, searching for key words (as above, e.g., [51,52]). Every attempt was made to be as inclusive as possible; however, in some cases, we were unable to retrieve some papers for use in this review.

### 2.2. Data Extraction

Only records on Diptera with aquatic larvae were extracted based on commonly used aquatic insect keys (e.g., [32]). From these, we extracted unique associations between aquatic Diptera and Zingiberales and Bromeliaceae taxa—defined as one Diptera taxon from one single plant taxon for one location. We include location data with GPS coordinates for each association when given in the source papers.

**Figure 3:**
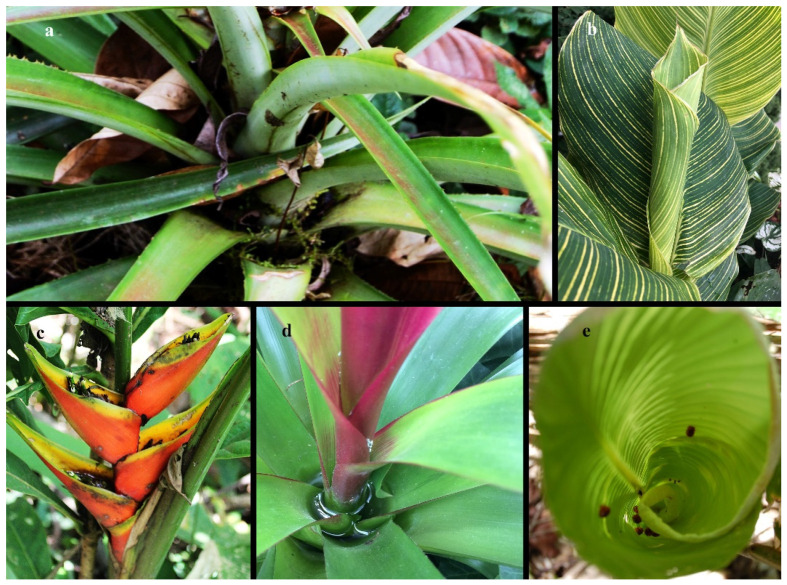
Phytotelma habitats of Bromeliaceae and Zingiberales: (**a**) lateral view of leaf axils, Bromeliaceae, *Ananas*, dorsal view (photo: C.S. Chaboo); (**b**) lateral view of leaf roll phytotelmata, Zingiberales, Marantaceae *Calathea*, potted plant, Kansas City, (photo: C.S. Chaboo); (**c**) inflorescence with bracts, Zingiberales, Heliconeacea, *Heliconia stricta* Huber, from Peru (photo: T. Förster); (**d**) dorsal view of leaf axils, Bromeliaceae, potted plant, Kansas City dorsal view (photo: C.S. Chaboo); (**e**) dorsal view of leaf roll phytotelmata, *Heliconia*, Trinidad, dorsal view (photo: C.S. Chaboo).

If available in the source material, we extracted data on the plant part containing the phytotelma. These data were inconsistently reported across the literature reviewed herein, so for the purposes of this paper, we define them as:

1. Bract—a modified leaf (scale-like or petal-like) subtending the flowers (or, later, the fruits). It is on the inflorescence (for an individual flower or a group of flowers) and may still persist on the infructescence.
2. Inflorescence—a group of flowers.
3. Infrutescence—a group of fruits, the next stage after pollinating the inflorescence.
4. Leaf axil—the cavity created by the leaf petiole or stalk that attaches the lamina to the stem.
5. Leaf roll—the flat photosynthetic leaf lamina, which is rolled into a cone when immature and holds water.
6. Leaf rosette or tank—water reservoir at the base of the leaves (Figure 3).

### 2.3. Database Construction

The database was constructed using MS Excel. The database (Supplementary Table S1) is arranged into 10 columns: plant family (Bromeliaceae or families within Zingiberales), plant genus or species, Diptera family, Diptera genus or species, phytotelma habitat location (e.g., leaf roll or bract), country location, collection site location, latitude and longitude for the collection site location, and citation for the source material from which these data were extracted. We denoted missing data as “undetermined”, meaning that either the original source material did not provide the data or we were unable to find the data in the source material.

### 2.4. Data Standardization

A small subset of assembled papers listed morphospecies (e.g., *Aedes* sp. 2, *Polypedilum* sp. 3). Although morphospecies are useful for estimating biodiversity within a study, they should not be compared across studies since there is seldom a way to match up morphospecies. For example, one research group’s *Aedes* sp. 2 may not be the same as another research group’s *Aedes* sp. 2. Thus, due to the taxonomic uncertainty of morphospecies in a large review, we did not include them as morphospecies; rather, we standardized morphospecies by listing them as their parent taxon (e.g., *Aedes* sp. 2 converted to *Aedes*) and so removed redundancies.

Taxa listed as “genus sp.” or “genus spp.” were standardized to “genus” for consistency across the database. Species “groups”, “complexes”, or “systems”, or groups were uniformly changed to species group, capitalizing the species name and the term group to clarify that the taxon listed was not that particular species, but instead was part of a group (e.g., *Culex* (*Culex*) *coronator* group was converted to *Culex* (*Culex*) Coronator Group). When publications listed both a species and its containing genus separately, we interpreted this to mean that the containing genus was different from the species listed and retained those data in the database.

### 2.5. Taxonomic Updates

Plant taxonomic nomenclature was updated according to the Global Biodiversity Information Facility [67] and the WFO Plant List [61]. Diptera classification and taxonomic nomenclature were updated using the Catalogue of Craneflies of the World (ccw.naturalis.nl, [68]), the Catalogue of Life ([69], http://catalogueoflife.org, last accessed on 15 October 2025), Systema Dipterorum ([70], http://www.diptera.org/, last accessed on 15 October 2025), the Global Biodiversity Information Facility [67], and the Mosquito Taxonomic Inventory [71]. We followed the global Diptera classification of Pape et al. [72], Evenhuis and Pape [70], and the Manual of Central American Diptera [73]. For some families, we further refined the classification based on the information contained in the websites listed above. Additionally, we followed Gelhaus and *Podeniene* [74], who split Tipulidae into the families Cylindrotomidae, Limoniidae, Pediciidae, and Tipulidae. Taxa assigned to Tipulidae prior to 2019 are collectively assigned to the superfamily Tipuloidea unless otherwise noted.

### 2.6. Location Updates

Geographic coordinates were updated into degree–minute–second (DMS) format and converted to decimal degrees for consistency and analysis. Directional indicators (N/S/E/W) were used to assign the correct sign to each value (i.e., negative for South and West).

### 2.7. Quality Assurance Protocols

We conducted multiple reviews of the database. We also closely examined 10% of data lines randomly selected, twice, by two different co-authors. We request that researchers and reviewers contact us with corrections, missed publications, and additional data.

### 2.8. Vector Analysis

Vector species were identified from [46,47,75,76] and compared with the compiled data extracted from the literature reviewed herein.

### 2.9. Data Analysis and Visualization

All data analyses and graphs were prepared using R version 4.4.2.

## 3. Results and Discussion

### 3.1. Literature Review

The literature from 1913 to 2025 and online data review yielded 173 papers with records of aquatic Diptera in Zingiberales and Bromeliaceae phytotelmata (Table 1, Supplementary Table S1). We organized the papers into four broad categories: Ecology, Survey, Taxonomy, and Other (Table 1). Ecology, Survey, and Taxonomy papers were further subdivided and shall be discussed further below.

Data were extracted from papers focused on phytotelmata from 44 countries in five biogeographic regions located across Asia, Africa, Australia, the Caribbean, Central America, North America, and South America (Table 2). Trinidad and Tobago had the most associations (Supplementary Table S1), reflecting surveys and mosquito surveillance programs (see these and related surveys, [51,52]). Gaps exist in geographic coverage (Table 2); the majority of documented associations are from the Neotropics, with far fewer studies published from the Australian, Afrotropical, or Oriental zoogeographic regions (Table 2). Aquatic Diptera in phytotelmata have received limited ecological research, with the exception of Culicidae (e g., [36,77]). Broader macroinvertebrate communities in phytotelmata have been studied extensively.

### 3.2. Surveys

We extracted the majority of unique associations between aquatic Diptera and bromeliad and Zingiberales phytotelmata from surveys. We define surveys as collection-based studies that targeted multiple taxa over a defined geographic range for phytotelmata in general or for specific plants that have phytotelma habitats or surveys of a single taxon of aquatic Diptera over a defined geographic range or from phytotelmata in general or for specific plants that have phytotelma habitats (46%, Table 1). Some of the earliest published surveys are from the early 1900s [5,13]. Surveys of bromeliad and Zingiberales phytotelmata have been conducted throughout the Neotropics, including Argentina, Brazil, Venezuela, the Caribbean islands, the Afrotropics (e.g., Tanzania), and the United States of America [33,34,37,78,79,80,81,82,83,84,85]. Surveys of specific habitats or regions include the Western Ghats, India [86], cloud forests in Venezuela [87], coffee-growing regions in the Colombian Andes [88], and the Everglades, Florida, USA [35].

Most taxon-specific surveys of aquatic Diptera we found in the literature focused on Culicidae, due to their medical significance, as indicated above. Some surveys aimed to identify the natural tank habitat of possible vector mosquitoes (e.g., [89]). The largest body of taxonomic survey research comes from a systematic review of collections from Central and South American Culicidae (e.g., [51,52,90,91,92,93]). Surveys and taxonomic research focused on other aquatic dipteran families included Ceratopogonidae [94,95,96,97,98], Chironomidae [99,100,101,102,103,104,105,106,107,108], Psychodidae [13], Statiomyidae [109], Syrphidae [110,111,112,113,114], and Tabanidae [115]. These studies pertained directly to aquatic Diptera in phytotelmata or included taxa from phytotelmata as part of larger taxonomic research.

Generalist and specialist species of Diptera have been documented from phytotelmata (e.g., [116]). Species may exhibit a specificity for either *Heliconia* or bromeliads [117]. Frank et al. [36] noted that two culicid species, *Wyeomyia* (*Wyeomyia*) *vanduzeei* Dyar & Knab and *Wyeomyia* (*Wyeomyia*) *mitchellii* (Theobald), have rarely been found in habitats other than bromeliad leaf axils. Taxonomic treatments and surveys documented many obligate species (e.g., [5,13,103,118,119,120]) but the lack of species-level taxonomic resolution in some surveys and ecological studies may hinder our understanding of the variables that drive species specificity for phytotelma habitats. Our own survey [57] focused on Diptera in general. Overall, we found that surveys are the most important tool in increasing knowledge of diversity (e.g., [121]).

### 3.3. Ecology

A relatively high percentage of papers we used to extract data on aquatic Diptera from phytotelmata focused on ecology (28%, Table 1), from many of which we extracted data for this study. Our review revealed that the small physical dimensions of phytotelmata strongly restrict the membership and complexity of the food web that develops relative to larger ecosystems (e.g., [21,116,122,123]).

Phytotelmata water volumes are small, with a watery pool and shallow margins with a thin wet film (hygropetic), thus resulting in the development of spatial heterogeneity. Bromeliad phytotelmata may hold up to nearly 1000 mL of water (e.g., [124]) or greater [125], and *Heliconia* bract pools may hold up to ~20 mL of water [80]. These volumes fluctuate based on rainfall amount and plant secretions [8,126], impacting community structure and function [23,124,127]. Water volume and patterns of precipitation have been linked to the abundance of the invertebrate community [36,128].

Phytotelma habitats change structurally depending on forest dynamics. For example, in the natural dynamics of forest habitats, tree falls and canopy gaps can shift local humidity and sunlight, increasing phytotelma habitats for some species of plant [29], an impact seen under anthropogenic changes as well [129]. Most importantly, these ephemeral microecosystems change rapidly over time. After initial inputs of water, typically by rainfall or secretion [8], the water is clear and nutrient-poor, supporting few organisms. Over time, decomposing organic material and detritus can alter water volume, quality, oxygen levels, and chemistry [9]. The accumulation of this fine particulate organic matter serves as nutrients for some organisms, which in turn become food for others. Thus, organic matter may increase diversity, but eventually concentrations increase enough to drive down concentrations of dissolved oxygen, negatively impacting diversity [116]. Organic matter opens niches for detritivores and algae, forming the base of many phytotelma food webs [103,130,131]. Organismal diversity may also reflect seasonal conditions (periods of high rainfall versus drier periods) and temporal niche partitioning [117]. The host plant can also influence the habitat, e.g., nutrients exudates have been reported in certain Zingiberales inflorescences [126]. Phytotelmata food webs show top-down and bottom-up effects driven by predators or detritivores, shredders, and scrapers [77,132] and possibly influenced by habitat size [18]. Communities may be consistent from year to year and within a plant but still vary based on phytotelma age and invertebrate taxon [117]. The diversity of invertebrates in phytotelmata is relatively low by taxa, biomass, and number of ecological roles (e.g., [103]).

Increasingly unpredictable and extreme climate conditions (e.g., hurricanes, intense rainfall, and drought) may exacerbate declining biodiversity. Phytotelma habitats can help buffer unpredictability and disturbance to maintain species presence, possibly acting as freshwater refugia during dry seasons [9,29]. Tight clusters of phytotelma-bearing plants allow for some insects, such as predators, to move between habitats during drought, but even isolated phytotelmata can help to maintain diversity during drought [133]. Furthermore, phytotelmata maintain an internal temperature that may buffer short-term swings of external temperature [134]. Everly and Yee [135] simulated hurricane conditions on *Heliconia* phytotelmata and found that diversity decreased in macroinvertebrate communities. A larger field study found that phytotelma community alpha and gamma diversity decline post hurricanes, likely due to a loss of habitat, but this change varied within and between communities [64], such that rare species declined, whereas common species remained stable. Srivastava et al. [136] found that simulated extreme rainfall and drought affected invertebrate functional composition but had little effect on overall biomass. Their results were confounded by the taxonomic composition across the broad geographic scale of their study—they did not use lower taxonomic resolution for their functional trait analysis, possibly impacting the outcome of their study. For example, Poff et al. [137] cautioned that the use of a family-level resolution in trait-based analysis may result in the loss of important information for families that exhibit a high diversity of traits. Chironomidae are listed as detritivores, filter gatherers, or predators [138], but taxa such as *Stenochironomus atlanticus* Pinho & Mendes [139], a Chironomini found in phytotelmata and a leaf miner, may be locally abundant. Thus, assigning traits at the chironomid family level reduces valuable information on food web relationships and functional ecology.

Insect larvae are difficult to identify to genus and species, and sometimes to the family level, in ecological studies. Most insect species are described based on the adult stage, and keys to larvae are lacking or difficult to find. One exception is aquatic Diptera. Mosquito identification keys are becoming more widely available (e.g., [48]), and keys to Diptera larvae can be found [32,73]. However, this difficulty in identifying larvae to genus or species is reflected in our results, with nearly 10% of the taxa identified only to the family level.

### 3.4. Taxonomy

Papers focused on the taxonomy of specific aquatic Diptera through revisionary work, phylogenetics, or biogeography composed 21% of the papers from which we extracted data for this study (Table 1). A total of 4979 unique associations between aquatic Diptera and phytotelmata formed by Bromeliaceae and Zingiberales were documented (Supplementary Table S1). Specificity of the unique associations in the data ranged from low-information content, plant family/Diptera family/general site descriptions to high-information content, plant species/Diptera species/phytotelma habitat/latitude and longitude.

#### 3.4.1. Plant Taxonomy

**Figure 4:**
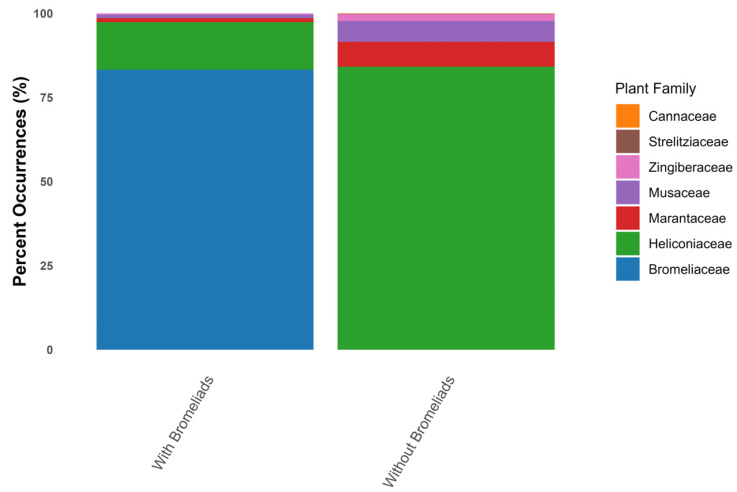
Percent occurrence of plant families for the unique associations between aquatic Diptera and phytotelmata for all families based on the entire set of unique associations and for only the families of Zingiberales. Note that Cannaceae and Strelitziaceae were included in the analysis but are present at such low numbers they do not appear on the graph.

Our research uncovered aquatic Diptera associated with phytotelmata from 117 species within 20 genera in one family (Bromeliaceae) in the order Poales and 6 families with 27 species in 10 genera in the order Zingiberales (Table 3). Bromeliaceae accounted for the greatest percentage of unique records between aquatic Diptera and phytotelmata in this study (Figure 4) when including all plant families. Heliconiaceae, followed by Marantaceae and Muscidae, accounted for the greatest percentage of unique records between aquatic Diptera and phytotelmata in Zingiberales (Figure 4). The plant families retrieved from the literature for our review are listed below.

**Bromeliaceae** (3740 named species, globally, WFO Plant List [61]). Data on 117 species from 26 genera were extracted from source material for this review (Table 3). Literature reporting associations of Diptera from phytotelmata were far more common for Bromeliaceae than the six families of Zingiberales (83%, Table 3). *Vriesea* Lindl. had the most species associated with Diptera, followed by *Aechmea* Ruiz & Pav. and *Tillandsia* L. within Bromeliaceae (Table 3).

**Cannaceae** (13 named species, globally, WFO Plant List [61]). No species within *Canna* L. have been reported with Diptera associates (Supplementary Table S1). *Canna* species grow up to 3 m. Many varieties have been developed due to their significance in gardens and agriculture [140].

**Heliconiaceae** (209 named species, WFO Plant List [61]). Diptera data on 16 species in 1 genus (*Heliconia*, commonly called heliconias) were extracted from source material (Table 3). *Heliconia* have large, broad leaves (Schultes [141]); new leaves open as upright, elongate leaf rolls where rainwater accumulates in this slowly opening cone. Fluid in bracts is actively maintained according to Bronstein [142] (*Heliconia imbricata*). Heliconiaceae phytotelmata are the most commonly studied in Zingiberales since the pioneering research of Seifert and Seifert [59] and numerous subsequent studies (e.g., [55,57,143,144]).

**Marantaceae** (585 named species, globally, WFO Plant List [61]). Data on 10 species in three Diptera genera were extracted from publications (Table 3). Marantaceae is commonly called the arrowroot or prayer-plant family because the leaves move, folding together at night. Like Heliconiaceae, the young leaves form leaf rolls that hold water. The floral bracts hold transparent fluid, which may be actively maintained [142]. Phytotelma communities of several *Calathea* G. Mey. species have been studied (e.g., [19,55,57,145]). Diptera in *Hylaenthe* Jonker was studied [57].

**Musaceae** (95 named species, globally, WFO Plant List [61]). Data on two species from *Musa* L. were extracted from the source material (Table 3). These tall herbs are native to Africa and Asia but are cultivated widely outside the native range for food (bananas, plantains, pseudostem, and flowers), starch, and textiles [146,147].

**Strelitziaceae** (12 named species, globally, WFO Plant List [61]). Data on one species were extracted from the source material (Table 3). These plants can grow up to 40 m tall, with leaves about 4 m long. The *Ravenala* Adans. phytotelmata are indeed axillary as they form in the long leaf stalks of these large plants. The widely used common name “traveller’s palm” refers to the folklore that thirsty travelers accessed the large volume of water in this phytotelmata (e.g., [148]).

**Zingiberaceae** (1908 named species, globally, WFO Plant List [61]). Data on three species from three Diptera genera were extracted (Table 3). Plants of the ginger family can grow up to 6 m. Well-known members are ginger, turmeric, and cardamom [149,150].

**Differences between phytotelmata habitats of Bromeliaceae and Zingiberales.** In both the information gleaned from the literature and our field experiences with these plants, we can discern differences in origin, structure, life span, and ecological functions, which are likely to influence the type of community that forms. The compartmentalized tank or vase of Bromeliaceae is large in space and water volume. Each leaf offers hygropetric and dry surfaces, providing more surface area to colonizers. Functionally, the tank provides nutrients (soil + organic decay) to the plant, so the plant may maximize habitat for organisms that enrich the pool. Bromeliads occur at multiple levels within a habitat, terrestrial to epiphytic, and this vertical stratification may influence the ecosystem membership. In contrast, the three types of Zingiberales phytotelmata—bract, leaf rolls, and (rarely studied) axils— are different from each other and from Bromeliad phytotelmata. All three are small, hold minute water volumes, and have shorter life spans. Bract pools are more open, with a wider surface; the multiple bracts on a long-lived inflorescence offer a unique chronosequence of mini-ecosystems, the youngest in newly opened bracts at the apex and older pools at the base [34,117,135,151]. The tightly rolled leaf (like a scroll) offers a large area of thin-film, perhaps even functioning as a biofilm; taxa living here are highly adapted for tight spaces (well-known are the highly flattened larvae and adults of certain Chrysomelidae beetles). The leaf rolls have a far shorter existence than bromeliad or Zingerales bract phytotelmata—a few weeks versus 1+ years (personal observations). The life span of the pool can influence whether colonizing organisms are short-term transients or longer-term inhabitants.

#### 3.4.2. Diptera Taxonomy

**Figure 5:**
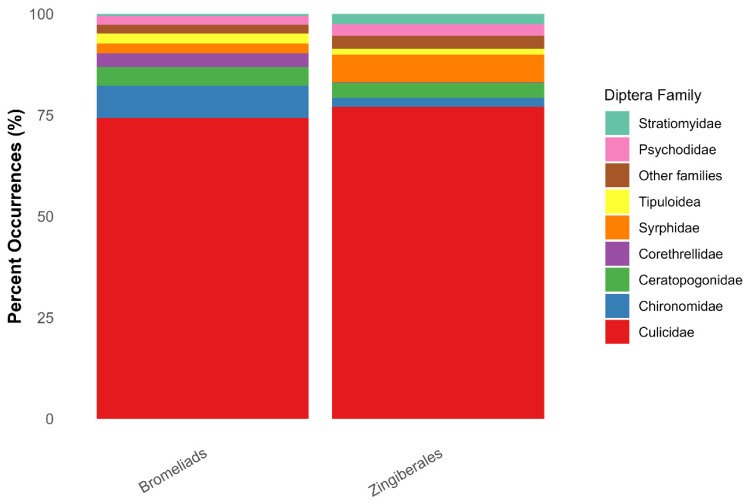
Percent occurrence of major aquatic Diptera families comparing those found in phytotelmata of Bromeliaceae and Zingiberales.

Fourteen families of aquatic Diptera were associated with phytotelmata in Bromeliaceae and/or Zingiberales (Table 4) across the 4979 unique associations, 479 of which were only identified to family level (Supplementary Table S1). Culicidae were the most common family documented from both Bromeliaceae and Zingiberales, while proportions of Stratiomyidae and Syrphidae increased in Zingiberales (Figure 5).

**Figure 6:**
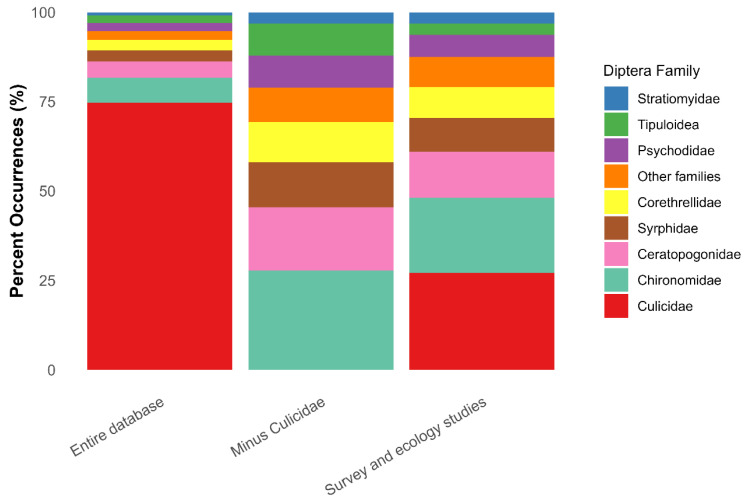
Percent occurrence of aquatic Diptera families for the entire set of 4979 unique associations, for all unique associations minus Culicidae, and for only papers that included surveys and ecology.

Culicidae were the most commonly documented family in the literature, comprising nearly 75% of unique occurrences in the literature when considering the entire database (Figure 6). The percentage of unique associations between Culicidae and phytotelmata families, genera, or species decreases to below 30% when excluding papers that focused only on Culicidae and instead included general surveys and ecological research (Figure 6). Ecological studies and community surveys that included data on a wide range of taxa may best represent the percentage of Culicidae within these habitats (Figure 6). Examining the entire database/all papers but simply removing all references to Culicidae clarifies the relative occurrences within the other families of aquatic Diptera (Figure 6).

The most common genera for Culicidae and hence the entire database were *Wyeomyia* Theobald, *Culex* Linnaeus, *Aedes* Meigen, *Toxorhychites* Theobald, and *Anopheles* Meigen (Supplementary Table S1). Excluding Culicidae, the most common genera in the database were *Corethrella* Coquiette (Corethrellidae), *Forcipomyia* Meigen (Ceratopogonidae), *Copestylum* Macquart, *Quichuana* Knab (Syrphidae), and *Monopelopia* Fittkau (Chironomidae). Chironomidae had the highest number of genera extracted from the source papers, followed by Culicidae, Ceratopogonidae, Psychodidae, and Syrphidae (Table 4). Culicidae was the most species-rich aquatic Diptera in the literature reviewed for this study, followed by Syrphidae, Chironomidae, Ceratopogonidae, and Psychodidae (Table 4). All families had fewer species associated with phytotelmata formed by Zingiberales than by bromeliads (Table 4). The most common families of aquatic Diptera in this study are listed below.

**Ceratopogonidae** (6700 named species, globally, [70]). Ceratopogonidae are common inhabitants of phytotelmata represented by 31 named species in 7 genera (Table 4). At least nine species appear to be obligate phytotelma dwellers, including one subgenus: *Culicoides heliconiae* Fox & Hoffman, *Forcipomyia* (*Phytohelea*) *bromelicola* (Lutz), *Forcipomyia* (*Phytohelea*) *caribbeana* Saunders, *Forcipomyia* (*Phytohelea*) *edwardsi* (Saunders), *Forcipomyia* (*Phytohelea*) *jocosa* Saunders, *Forcipomyia* (*Phytohelea*) *keilini* (Saunders), *Forcipomyia* (*Phytohelea*) *magna* (Saunders), *Forcipomyia* (*Phytohelea*) *oligarthra* Saunders, *Forcipomyia bromeliae* Saunders, *Forcipomyia calatheae* Wirth (Supplementary Table S1).

Ceratopogonidae are predominantly aquatic, commonly found in smaller aquatic habitats such as phytotelmata [30]. They play a role in cacao pollination [152], leading to studies of bromeliads as nurseries for larvae of adult pollinators of cacao [96,153]. Some adults vector diseases, such as blue tongue, to livestock [154]. Ceratopogonid larvae exhibit cuticular respiration and may undulate, moving their body back and forth to increase the flow of dissolved oxygen across their thin larval cuticle [49]. Ceratopogonid respiratory adaptations allow them to colonize extreme environments with low concentrations of dissolved oxygen (e.g., [155]), such as are found in some phytotelmata (e.g., [16]). Larval ceratopogonids have various functional roles in aquatic communities, including collector-gatherers and predators [32]. The larvae are small and difficult to collect and identify, which may have led to a collection bias in terms of their documented diversity [30]. Thus, the known biodiversity of ceratopogonids from phytotelmata would increase with taxon-specific sampling and identifications by expert taxonomists.

**Chironomidae** (8425 named species, globally, Courtney [156]). Our data extraction revealed that chironomids are common in phytotelmata from Bromeliaceae and Zingiberales, represented by 37 named species in 26 genera (Table 4). At least seven species appear to be obligate phytotelma-dwellers, including *Phytotelmatocladius delarosai* Epler, *Tanytarsus bromelicola* Cranston, and *Monopelopia tillandsia* Beck & Beck, *Monopelopia mikeschwartzi* Epler, *Monopelopia caraguata* Mendes, Marcondes, and de Pinho, and *Polypedilum* (*Polypedilum*) *panacu* Mendes, Andersen, and Jocqué (Supplementary Table S1) [103,105,108,120]. *Chirocladius pedipalpus* Picado [5] seems to be valid still, although based on the illustration in Picado [5] and the discussion in Cranston and Judd [101] and Sæther et al. [157], it is a species of *Polypedilum*.

Chironomidae are a ubiquitous family of aquatic Diptera, found in nearly every type of aquatic ecosystem [158], and are common in temporary waters [159], particularly in phytotelmata where they maintain breeding populations [8]. Although less studied than Culicidae, chironomid diversity and distribution may be as great or greater in phytotelmata [120]. Chironomid larvae exhibit cutaneous respiration and, like Ceratopogonids, may undulate to increase the flow of dissolved oxygen across their thin cuticle, increasing respiration [49]. A subgroup of Chironomidae is known as blood worms for the red pigment carried in their complex hemoglobins. These hemoglobins facilitate the uptake of oxygen, allowing chironomids to colonize environments with extremely low concentrations of dissolved oxygen [49,160], such as the low-oxygenated environment found periodically in phytotelmata (e.g., [16]). Chironomidae are diverse, filling a variety of functional roles in aquatic ecosystems [138]. Taxonomic resolution and ecological knowledge are improved when ecologists collaborate with taxonomic experts, resulting in reliably identified chironomid genera, including *Chironomus*, *Metriocnemus*, *Monopelopia*, *Polypedilum*, and *Tanytarsus* [120].

**Corethrellidae** (138 named species, globally, Courtney [156]). Corethrellidae were common inhabitants of phytotelmata based on data extracted from source publications for this study, represented by one genus and four named species (Table 4).

We detected no obligate phytotelma-dwelling species in this review (Supplementary Table S1). This family has low diversity relative to the high number of associations with phytotelmata documented in the literature, perhaps reflecting the overall low diversity in the family [161]. One publication listed Chaoboridae (phantom midges) as present in phytotelmata, but we consider this unlikely, given that chaoborids typically live in larger open bodies of water. The genus *Corethrella* was part of Chaoboridae until elevated to the family level by Wood and Borkent [162], and so the report most likely relates to Corethrellidae rather than Chaoboridae. Borkent [163] noted that many species await discovery and description, and this is likely true for those that live in phytotelmata as well. Larvae and pupae of *Corethrella*, the only genus in this family, are unknown for over half of the currently documented species [161]. Functional roles of larvae include predation [32].

**Culicidae** (3777 named species, globally, Courtney [156]). Culicidae are the most common aquatic Diptera in phytotelmata of Bromeliaceae and Zingiberales, represented by 227 named species in 21 genera (Table 4). Although we were unaware of any obligate phytotelma-dwelling species of culicids by their scientific names, Frank et al. [36] suggested that *Wyeomyia* (*Wyeomyia*) *vanduzeei* and *Wyeomyia* (*Wyeomyia*) *mitchellii* may be obligate phytotelma-dwellers. The high number of named species we were able to extract from the literature reflects that many specimens were reared to associate adults with larvae so that species names could be assigned. Given their significant diversity in phytotelmata, it is likely that some obligate culicid species await discovery and documentation from Bromeliaceae and Zingiberales.

Culicid larvae are found in habitats ranging from streams, wetlands, and natural and human-made container habitats [164]. Some culicid genera (e.g., *Aedes* and *Culex*) are common in natural containers, such as phytotelmata [164]. Culicidae are adapted to survive in low-oxygenated environments by breathing atmospheric oxygen using respiratory siphons and have short life cycles, so are not limited by ephemeral habitats [50], such as phytotelmata. The larvae move up and down the water column and so may function to circulate the small amount of settled detritus throughout the phytotelma habitat. Culicid larvae functional roles include collector-gatherers and filterers [50]; some have important roles as predators in phytotelma ecosystems [165].

Some Culicidae species are important vectors for diseases, including malaria, Dengue fever, Oropouche virus, Zika, West Nile, and different types of encephalitis [151,164]; thus, their presence in phytotelmata is of concern and spurred large geographic surveys, such as Belkin and Heinemann’s [91] ‘Mosquitoes of Middle America’. The relatively low percent of vector species and low percent of vector species/phytotelmata associations (Table 5) indicates that caution should be used when advocating for the removal of the plants containing phytotelma habitats (e.g., [166]). Nineteen species of Culicidae were identified as vectors of human disease based on the data extracted from publications for this study (Table 5), representing only a small percentage of aquatic Diptera/phytotelmata associations (6%) reported herein. Vectors comprised a small percentage (8%) of Culicidae/phytotelmata associations reported from studies focused only on Culicidae. *Anopheles* species are of the greatest concern for their role in vectoring malaria and accounted for the highest proportion of vector mosquitoes in the study (Table 5).

**Psychodidae** (3402 named species, globally, Courtney [156]). Psychodidae larvae are both terrestrial and aquatic. Worldwide, approximately 2000 species are aquatic, mostly from the subfamily Psychodinae [30]. Psychodidae have not been commonly reported in the literature and were represented by only 13 named species in 7 genera in this review (Table 4). Most of the associations we have extracted from the literature were for undetermined psychodids or genus-level identifications (Supplementary Table S1). At least one species is an obligate phytotelma-dweller, and two others have been described from Bromeliad phytotelmata: *Neurosystasis bromeliphila* Wagner & Hribar, *Moruseodina cusucoensis* Bravo & Cordeiro, and *Psychoda romeroi* Bravo, Lopes & Bastos [167,168,169].

Recent studies and surveys have increased the number of species from phytotelmata of bromeliad and Zingiberales [167,168,169], indicating that targeted sampling may increase the known diversity of this taxon from phytotelmata. Larvae of aquatic Psychodidae are difficult to identify to genus. Courtney’s [32] keys to larvae in North America backed off some of the earlier genus designations, leaving the taxa at Pericomaini and Psychodini. Larvae are not presented in the Manual of Central American Diptera keys [73]. Larvae of aquatic Psychodidae may use respiratory tubes with apical hydrofuges to breathe atmospheric oxygen when in low-oxygenated aquatic environments; the hydrofuges allow them to hang off the surface tension of water, facilitating their respiration [49]. Psychodid larvae function as collector-gatherers in aquatic ecosystems [32].

**Stratiomyidae** (3402 named species, globally, Courtney [156]). Only 43 species of Stratiomyidae are aquatic in their immature stages [30]. The few stratiomyid larvae documented from bromeliad and Zingiberales phytotelmata were represented by four named species in five genera (Table 4). No obligate phytotelma-dwelling stratiomyids were found through our search of the literature.

Like Psychodidae, aquatic Stratiomyidae larvae also use a hydrofuge to hang off the surface tension of water so that they may breathe atmospheric oxygen through an anal respiratory tube of varying shapes and sizes [30,49]. This adaptation allows aquatic stratiomyid larvae to colonize low oxygen environments, such as thermal springs (e.g., [170,171,172]) and phytotelmata (e.g., Bromeliaceae, [18]). Stratiomyid larvae generally function as collector-gatherers in aquatic ecosystems [32]. Stratiomyidae larvae are seldom found in phytotelmata; however, the percentage of recorded associations we extracted from the source material, particularly for Zingiberales (Figure 6), indicates that more thorough surveys of phytotelmata involving taxonomic experts will reveal greater diversity of Stratiomyidae in the future.

**Syrphidae** (6566 named species, globally, Courtney [156]). Less than a quarter of species are aquatic [30], but notable habitats for larval aquatic Syrphidae include phytotelmata, particularly bromeliads [30]. Syrphids were represented by 46 named species in 7 genera (Table 4). At least two obligate phytotelma-dwellers are identified by name in the literature: *Quichuana bromeliarum* Ricarte & Marcos-García and *Quichuana calathea* Shannon. Possibly, *Quichuana picadoi* Knab is also an obligate phytotelma-dweller (Supplementary Table S1).

Larvae, called rat-tailed maggots in some parts of the world, are adapted to low oxygen environments due to their long, rat-tail-shaped respiratory tube. Like Culicidae, Psychodidae, and Statiomyidae, syrphid larvae use their respiratory tube to breathe atmospheric oxygen [49] and are not dependent on dissolved oxygen for respiration. Aquatic syrphid larvae generally function as collector-gatherers [32] but may also be predators. Relatively recent surveys of Syrphidae described 22 new species, described from bromeliads [111,113], suggesting that future targeted surveys are likely to increase the known diversity of Syrphidae from phytotelmata.

**Tipuloidea** (15,733 named species, globally, Oosterbroek [173]). Two families within Tipuloidea have been reported in the literature we reviewed. Most species are aquatic or semi-aquatic as larvae [174].

Limoniidae (10,804 named species, globally, Oosterbroek [173]). Limoniids were represented by three named species in two genera (Table 4). Two species appear to be obligate in bromeliad phytotelmata: *Trentepohlia* (*Paramongoma*) *bromeliadicola* Alexander, *T.* (*P.*) *dominicana* Alexander, and *T.* (*P.*) *leucoxena* Alexander [118,119].

Tipulidae (4364 named species, globally, Osterbroek [173]). Tipulidae larvae have rarely been found in bromeliad and Zingiberales phytotelmata, and the family is represented by one genus (Table 4).

Some larval Tipuloidea tolerate low-oxygenated environments, such as phytotelmata, by breathing atmospheric oxygen through complex lobes with hydrofuge surrounding anal spiracles [74], whereas other larval Tipuloidea respire via cutaneous respiration [49]. Larvae of aquatic Tipulidae and Limoniidae fill numerous functional roles as collector-gatherers, shredders, and predators [74]. Given the large diversity in Tipuloidea, it is likely that additional obligate phytotelma-dwelling species await discovery.

**Other Families.** The remaining Diptera families found in bromeliad and Zingiberales phytotelmata were relatively rare (Table 4), represented by five families, with four species in four genera (Table 4). These families have few aquatic species, but they may be collected in streams, lakes, and streams worldwide [30].

### 3.5. Conservation

The availability and ubiquity of phytotelmata of Bromeliaceae and Zingiberales within tropical-subtropical habitats increase the heterogeneity of microhabitats and amplify biodiversity (taxonomic, ecological, and behavioral). Larger-scale anthropogenic changes resulting in habitat degradation and land-use changes alter local and regional biodiversity, potentially with extinctions of indigenous species, invasions of non-native species, and weakening of species-interaction networks [175,176]. The removal of epiphytic bromeliads, due to the mistaken belief they are parasites harming desired trees, is not advised since these bromeliads provide pollinator habitats for some tree crops (e.g., fly pollinators of cocoa trees [177]) or attract predators of damaging herbivores [178,179]. On the other hand, the introduction of attractive bromeliads and heliconias in gardens and public spaces far outside their native range provides habitats for local and invasive species [36].

Bromeliads provide many ecosystem services, such as provisioning, regulating, supporting, and cultural services [180]. Food, water, climate regulation, decomposition, and water filtration are important in supporting microecosystems, such as phytotelmata. The aquatic insects in phytotelmata also provide ecosystem services via the export or exchange of energy between the aquatic and terrestrial ecosystems (e.g., [41]), particularly as a food source for other animals. Habitat loss also diminishes these services to the point that Rosa et al. [129] suggested that phytotelma-faunal diversity and/or altered species composition may be useful as environmental indicators of impact from forest fragmentation, agriculture, and invasive species.

Phytotelmata are often perceived as habitats for mosquitoes that vector disease, which can lead to management strategies that remove even native bromeliads (e.g., [89]). While vector mosquitoes pose significant health risks to humans and livestock, they generally comprise a minor proportion of phytotelma communities [181]. *Anopheles* (*Kerteszia*) species, common Malaria vectors, are found frequently in bromeliads, leading to the concept of “bromeliad malaria” [43]. Contrary to the assumption that removal of forest and bromeliads near human habitation will decrease these mosquito vectors, Multini et al. [166] found that decreasing bromeliad diversity may lead to an increase in mosquitoes seeking human hosts. Natural hosts in the forest decrease with forest/habitat loss and fragmentation, leading the mosquitoes to seek blood meals from humans and increasing the potential for infection. Diverse macroinvertebrate communities in phytotelmata likely support predators such as *Toxorhynchites* mosquitoes, which help control malarial vectors [165].

## 4. Discussion

### 4.1. Taxonomy

Specialist surveys at the order, family, or genus levels greatly expand known diversity, including aquatic Diptera in phytotelmata (e.g., [111,121]). In Peru, Hayford et al. [57] doubled records of aquatic Diptera Zingiberales phytotelmata by analyzing taxa from only a few specimens. Because Diptera larvae are difficult for non-specialists to identify to genus or species, collaborations with taxonomists and improved larval keys (e.g., [48]) are essential. Continued taxonomic focus can reveal more new species inhabiting phytotelmata, including obligate phytotelma-dwellers [105,108,182]. New species descriptions, particularly within revisionary research and reviews (e.g., [113,157]) that combine molecular sequences, can produce more comprehensive biodiversity information on aquatic Diptera in phytotelmata. Most of the studies reviewed herein are limited in geographic scope (Table 1, Figure 4, see [11,183]), leaving gaps in our knowledge of the distribution and endemism of phytotelma Diptera. Studies that have brought together taxonomic specialists to survey broader geographic regions have vastly increased known diversity [121].

**Recommendations**. From our review, we recommend the following to advance discovery:

1. Coordinate surveys across large geographic scales (countries, ecoregions, and biogeographic regions) and address geographic gaps. The Mongolian Aquatic Insect Survey [184] is an exemplary model of coordinated taxonomic experts documenting biodiversity and describing associated ecological conditions.
2. Collect exhaustively, as collecting for a single taxon may miss diversity. Phytotelmata sampling can be destructive, so we ought to collect comprehensively for future research.
3. Make greater efforts to identify specimens to the species level by rearing larvae to adults and/or using molecular techniques such as DNA barcoding or meta barcoding.
4. Compile keys to larvae and species descriptions, many cited in this paper.
5. Engage taxonomic expertise for non-target taxa as needed (see more below).
6. *A posteriori* analyses must accept that original researchers identified both host plant and inhabiting organisms accurately; thus, we recommend that research that includes species descriptions, even in ecological work, be accompanied by the creation of voucher records and the deposition of vouchers in museums and other biodiversity depositories.

### 4.2. Ecology

Our understanding of phytotelmata microecosystems has advanced from early limnological studies to large-scale datasets [16,29,136]. Initiatives like The Bromeliad Working Group [185] exemplify how coordinated research and open biodiversity data enhance research. Publication of data supporting ecological research, particularly in large tables, appendices, or databases, has improved our understanding of biodiversity from ecological research (e.g., [18]). Improving the resolution of Diptera taxonomy deepens our knowledge of their diversity in phytotelmata.

**Recommendations.** To advance biodiversity through ecological descriptive work and analyses, we recommend the following:

1. List taxa used in tables, appendices, and Supplementary Materials and include plant names, phytotelmata type (e.g., leaf axils, leaf rosettes, leaf rolls, bracts), and geographic coordinates.
2. Include taxonomic specialists in research teams to provide genus- and species-level identifications.
3. Consult taxonomic expertise, such as those with *Systema Dipterorum* [70].
4. When possible, ecological research in which taxa are identified should be accompanied by the creation of a voucher collection and the deposition of vouchers in museums and other biodiversity depositories.
5. Use standardized data formats and metadata conventions to facilitate data sharing and synthesis across studies (e.g., [18]).

### 4.3. Plant/Insect Interactions

Studying insects in phytotelmata examines plant/insect interactions. Our review extracted data from source materials and found unique associations (Supplementary Table S1). Plant secretions form part of the chemical structure of phytotelma habitats, particularly in Zingiberales plants (see [8] for review), and are an important part of food webs [29]. Our review focuses on aquatic Diptera diversity in these ephemeral microecosystems, though many sources lacked genus or species names for the Bromeliaceae or Zingiberales plants hosting the phytotelmata (Supplementary Table S1). Very few papers reviewed here included in their entirety phytotelma plant species names, the location of the phytotemata on the plant, and Diptera (and other faunal group) information by location within the phytotelmata (Supplementary Table S1). As threats increase to the plants (e.g., [65]), declining plant species will also drive declines in the phytotelma Diptera species.

**Recommendations**. To advance knowledge of phytotelmata/insect interactions:

1. Include both botanists and entomologists in surveys and ecological research for accurate identifications or consult experts post-sampling.
2. Record and report the location of phytotelmata in each plant in tables, appendices, and Supplementary Materials.
3. Investigate community variation across sub-habitats within phytotelmata (e.g., outer versus inner whirls of leaf rolls and leaf rosettes).

### 4.4. Threats and Conservation

Global declines in freshwater macroinvertebrates, including Diptera, are often due to a loss of habitat [2,3]. For phytotelma freshwater microecosystems, the decreasing diversity of the plants is inextricably linked to the loss of phytotelmata and subsequent loss of diversity that phytotelma habitats host (e.g., [64]). Even shifts in light intensity may alter the phytotelma habitat [186,187]. Removing bromeliads in public health campaigns to manage mosquito disease vectors [9] is a fraught strategy as it contributes to habitat loss and may increase the transmission of certain mosquito-vectored diseases, as seen with general deforestation [166].

**Recommendations.** While broader responses to deforestation and climate change are beyond the scope of this review, we recommend that researchers:

1. Analyze phytotelmata communities for vector prevalence and explore possible community management of vector mosquitoes to avoid the destruction of phytotelma plants, particularly bromeliads.
2. Communicate the ecosystem services of Diptera from phytotelma habitat (e.g., pollination, aquatic to terrestrial energy subsidies) to support conserving bromeliad and Zingiberales phytotelmata.
3. Work with or establish local citizen science programs to monitor phytotelma insect communities, thereby increasing awareness of the joy and wonder of these unique aquatic ecosystems.

## 5. Conclusions and Future Directions

This exhaustive review of over 100 years of published literature on aquatic Diptera in phytotelma habitats of Bromeliaceae and Zingiberales resulted in a database (Supplementary Table S1) that we used to summarize research, identify gaps in the research, and make recommendations to fill those gaps. The large focus on Culicidae in phytotelmata due to the role of mosquito vectors in deadly diseases may have obscured the diversity of other aquatic Diptera, as well as the diversity of non-vector Culicidae. Future research that focuses on taxonomic surveys targeting specific groups of aquatic Diptera is likely to increase known biodiversity in regions where phytotelmata are common. Furthermore, ecological and conservation research that includes Diptera, Bromeliaceae, and Zingiberales taxonomists will enhance our understanding of the species that inhabit these unique and ephemeral microecosystems. The database resulting from this review (Supplementary Table S1) should serve as an objective, informed tool and baseline for future studies.

## Figures and Tables

**Table 1:** Categories of papers reviewed related to phytotelmata in Bromeliaceae and Zingiberales. Data on aquatic Diptera were extracted from these papers.

Topic	Number of Papers
Ecology (28%)	
Biodiversity, climate change, conservation	6
Descriptive	29
Experimental and theoretical	14
Survey (46%)	
Community	32
Taxa	47
Taxonomy (21%)	
New species and species lists	26
Revisions, phylogenetics, biogeography	11
Other (5%)	
Parasite, endosymbiont associations, oviposition biology	8

**Table 2:** Zoogeographic regions, continents or regions, and countries represented by the 4979 unique associations of aquatic Diptera with phytotelmata in Bromeliaceae and Zingiberales. (%) indicates the percent of records for each biogeographic region.

Country	Continent/Region	Country	Continent/Region
Afrotropical (0.3%)		Neotropical (95%)	
Central African Republic	Africa	St Vincent/Grenadines	Caribbean
Nigeria	Africa	St. Lucia	Caribbean
Tanzania	Africa	Trinidad and Tobago	Caribbean
Uganda	Africa	UK: Montserrat	Caribbean
South Africa	Africa	USA: Puerto Rico	Caribbean
Oriental (1.2%)		USA: Virgin Islands	Caribbean
India	Asia	Costa Rica	Central America
Sri Lanka	Asia	Guatemala	Central America
Malaysia	Asia	Honduras	Central America
Singapore	Asia	Jamaica	Central America
Indonesia	Asia	Mexico	Central America
Australasian (0.2%)		Panama	Central America
Australia	Australia	Argentina	South America
Papau New Guinea	Oceana	Brazil	South America
Neotropical (95%)		Bolivia	South America
Antigua and Barbuda	Caribbean	Colombia	South America
Bahamas	Caribbean	Ecuador	South America
Cuba	Caribbean	Peru	South America
Dominica	Caribbean	France: French Guiana	South America
Dominican Republic	Caribbean	Guyana	South America
France: Guadeloupe	Caribbean	Suriname	South America
France: Martinique	Caribbean	Venezuela	South America
Grenada	Caribbean	Nearctic (3.4%)	
Netherlands: Saba	Caribbean	USA	North America

**Table 3:** The number of genera and named species identified from Bromeliaceae and families of Zingiberales based on the 4979 unique associations extracted from the published literature for this review.

Plant Family	Number of Genera	Number of Named Species
Bromeliaceae	26	117
Cannaceae	1	0
Heliconiaceae	1	15
Marantaceae	3	7
Musaceae	1	1
Strelitziaceae	1	1
Zingiberaceae	3	3

**Table 4:** The number of genera and named species of aquatic Diptera associated with Bromeliaceae and families of Zingiberales, based on the 4979 unique associations extracted from the published literature for this review.

	Total		Bromeliaceae		Zingiberales	
Family	Number of Genera	Number of Species	Number of Genera	Number of Species	Number of Genera	Number of Species
Ceratopogonidae	7	31	7	23	3	8
Chironomidae	26	37	26	37	3	3
Corethrellidae	1	5	1	5	1	U
Culicidae (20 vectors)	21	227	20	203	17	72
Dolichopodidae	1	U	U	U	1	U
Empididae	U	U	U	U	U	U
Ephydridae	U	U	U	U	U	U
Muscidae	1	U	1	1	U	U
Psychodidae	7	13	7	10	4	6
Stratiomyidae	4	5	2	2	3	3
Syrphidae	7	46	6	37	4	11
Tabanidae	2	4	2	4	U	U
Tipuloidea *	U	U	U	U	U	U
Limoniidae	2	3	2	3	2	2
Tipulidae	1	U	1	U	0	0

U indicates undetermined, as recovered from the original source material or our inability to determine the taxa. * Published as Tipulidae prior to separation of Tipulidae into separate families, such as Limoniidae in the Nearctic in 2019.

**Table 5:** The proportion of known mosquito vector species associated with Bromeliaceae and families of Zingiberales, based on the 4979 unique associations extracted from the published literature for this review. Proportions are listed for species based on all aquatic Diptera and for only the mosquito records.

Mosquito Vector Species	Proportion of All Aquatic Diptera	Proportion for Only Mosquito Records
*Aedes* (*Fredwardsius*) *vittatus* (Bigot)	0.06	0.08
*Aedes* (*Protomacleaya*) *triseriatus* (Say)	0.04	0.05
*Aedes* (*Rampamyia*) *notoscriptus* (Skuse)	0.02	0.03
*Aedes* (*Stegomyia*) *aegypti* (Linnaeus)	1.14	1.53
*Aedes* (*Stegomyia*) *albopictus* (Skuse)	0.82	1.10
*Aedes* (*Stegomyia*) *simpsoni* Theobald	0.16	0.21
*Aedes mediovittatus* (Coquillett)	0.02	0.03
*Anopheles* (*Anopheles*) *pseudopunctipennis* (Theobald)	0.02	0.03
*Anopheles* (*Kerteszia*) *bellator* Dyar & Knab	1.16	1.56
*Anopheles* (*Kerteszia*) *cruzii* Dyar & Knab	0.20	0.27
*Anopheles* (*Kerteszia*) *homunculus* Komp	0.70	0.94
*Anopheles* (*Kerteszia*) *neivai* Dyar & Knab	0.76	1.02
*Anopheles* (*Kerteszia*) *pholidotus* Zavortink	0.02	0.03
*Anopheles* (*Kerteszia*) Theobald	0.12	0.16
*Anopheles* (*Nyssorhynchus*) *argyritarsis* (Robineau-Desvoidy)	0.10	0.13
*Anopheles* (*Stethomyia*) *kompi* Edwards	0.04	0.05
*Culex* (*Culex*) *annulirostris* Skuse	0.02	0.03
*Culex* (*Culex*) *coronator* Dyar & Knab	0.12	0.16
*Culex* (*Culex*) *quinquefasciatus* Say	0.46	0.62
Total	6.01	8.03

## Data Availability

All data that were extracted from original source papers and compiled as part of this review are available at https://www.mdpi.com/article/10.3390/insects17030280/s1, Supplementary Table S1.

## Supplementary Materials

The following supporting information can be downloaded at: https://www.mdpi.com/article/10.3390/insects17030280/s1, Table S1: Unique associations between aquatic Diptera and phytotelma-habitat in Bromeliaceae and Zingiberales. References [90,99,122,188,189,190,191,192,193,194,195,196,197,198,199,200,201,202,203,204,205,206,207,208,209,210,211,212,213,214,215,216,217,218,219,220,221,222,223,224,225,226,227,228,229,230,231,232,233,234,235,236,237,238,239,240,241,242,243,244,245,246,247,248,249,250,251,252,253,254,255,256,257,258,259,260,261,262,263] are cited in the supplementary materials.
